# Polymer Encapsulated Liposomes for Oral Co-Delivery of Curcumin and Hydroxytyrosol

**DOI:** 10.3390/ijms24010790

**Published:** 2023-01-02

**Authors:** Vincenzo De Leo, Anna Maria Maurelli, Livia Giotta, Valeria Daniello, Sante Di Gioia, Massimo Conese, Chiara Ingrosso, Fulvio Ciriaco, Lucia Catucci

**Affiliations:** 1 Department of Chemistry, University of Bari Aldo Moro, Via Orabona 4, 70126 Bari, Italy; 2 Department of Biological and Environmental Sciences and Technologies, University of Salento, S.P. Lecce-Monteroni, 73100 Lecce, Italy; 3 Department of Medical and Surgical Sciences, University of Foggia, Viale L. Pinto 1, 71122 Foggia, Italy; 4 CNR-IPCF S.S. Bari, c/o Department of Chemistry, University of Bari Aldo Moro, Via Orabona 4, 70126 Bari, Italy

**Keywords:** liposome, curcumin, hydroxytyrosol, oral delivery, PEG, Eudragit S100, simulated digestion, TEAC, Caco-2 cells, MTT

## Abstract

Curcumin (Cur) is a hydrophobic polyphenol from the rhizome of *Curcuma* spp., while hydroxytyrosol (HT) is a water-soluble polyphenol from *Olea europaea*. Both show outstanding antioxidant properties but suffer from scarce bioavailability and low stability in biological fluids. In this work, the co-encapsulation of Cur and HT into liposomes was realized, and the liposomal formulation was improved using polymers to increase their survival in the gastrointestinal tract. Liposomes with different compositions were formulated: Type 1, composed of phospholipids and cholesterol; Type 2, also with a PEG coating; and Type 3 providing an additional shell of Eudragit^®^ S100, a gastro-resistant polymer. Samples were characterized in terms of size, morphology, ζ-potential, encapsulation efficiency, and loading capacity. All samples were subjected to a simulated in vitro digestion and their stability was investigated. The Eudragit^®^S100 coating demonstrated prevention of early releases of HT in the mouth and gastric phases, while the PEG shell reduced bile salts and pancreatin effects during the intestinal digestion. In vitro antioxidant activity showed a cumulative effect for Cur and HT loaded in vesicles. Finally, liposomes with HT concentrations up to 40 μM and Cur up to 4.7 μM, alone or in combination, did not show cytotoxicity against Caco-2 cells.

## 1. Introduction

Phenolic compounds represent an important class of secondary plant metabolites, characterized by one or more hydroxyl groups and by outstanding antioxidant properties. Depending on the chemical structure different classifications are possible, but generally they are divided into different groups such as phenolic acids, flavonoids, tannins, coumarins, lignans, stilbenes, quinones, and curcuminoids [1].

Numerous health-promoting effects are recognized in this class of compounds, which mainly derive from their antioxidant and anti-inflammatory properties. In fact, it has been shown that polyphenols can prevent numerous diseases such as cardiovascular and neurological ones, can counteract fungal, bacterial, and viral pathogens, and can even have antitumor activity [2,3].

For this reason, polyphenols are increasingly used in food supplements as natural food additives or functional food ingredients. Their therapeutic use as real drugs for the treatment of various diseases, including cancer, has also been proposed [4,5].

However, polyphenols have poor absorption and biodistribution, but also a fast metabolism and excretion in the human body, resulting in poor bioavailability which might hinder polyphenol in vivo effects [6].

Colloidal delivery systems represent an effective strategy to adequately protect and deliver polyphenols, preventing their degradation, increasing their stability in the biological environment and their uptake towards specific targets. In addition, the combination of different polyphenols, or polyphenols and other bioactive molecules, is considered a useful way to overcome the limitations related to poor bioavailability [4,7]. 

In this work, phospholipid-based liposomes were developed for the co-delivery of curcumin (Cur) and hydroxytyrosol (HT).

Cur is the main hydrophobic polyphenol extracted from the rhizome of *Curcuma* spp. and is endowed with numerous bioactive properties [8]. Together with its structural analogues, collectively known as curcuminoids, Cur exerts strong antioxidant, anti-inflammatory, antimicrobial and also anticarcinogenic properties [9], while showing low oral toxicity [10]. Cur has a very limited solubility in H_2_O [11], and undergoes fast degradation at neutral alkaline pH values of intestinal fluids. In addition, when it is orally administered, most of Cur is eliminated with feces. Therefore, the oral bioavailability of free Cur is very low, approximately 1% [12].

HT is a polyphenol mainly found in products of *Olea europaea*, especially in olive tree leaves, drupes and extra virgin oil [13]. HT and its derivatives, such as oleuropein and other secoiridoids, show high antioxidant capacity, with important bioactivity as a cardiovascular protector, neuroprotector and antiproliferative agent [14]. HT can prevent undesired lipid oxidation at the cellular level [15], and therefore could be used as a safe and natural food additive, cosmetic ingredient and medicine [16]. However, HT is very sensitive to air and light and its strong instability inevitably affects its biological activity. Its aqueous solutions darken quickly, probably due to oxidative degradation [3]. Additionally, HT may interact with proteins and other food nutrients, which may affect the extension of the metabolization and, therefore, its bioavailability in the body. Furthermore, although it appears to be stable under acidic conditions, during intestinal incubation free HT concentration progressively decreased in parallel with an increase in 3,4-dihydroxyphenyl acetic acid. After two hours an apparent loss of about 20% was observed, both due to the effect of digestive enzymes and the effect of the neutral alkaline conditions that characterize intestinal fluids (pH = 7.5) [17].

Liposomes are one of the most established systems among colloidal carriers for drug delivery due to their high biocompatibility, the relative simplicity of preparation and the versatility of application [18]. Liposomes are phospholipid vesicles capable of receiving and delivering molecules with different polarities, with a high possibility of stabilization, functionalization and targeting, for example, through the use of polymers and nanoparticles [18,19,20]. The lipid vesicles can protect their payload and introduce it into the cells through endocytosis. With their double compartment, liposomes lend themselves well to loading both Cur and HT. Cur can be accommodated in the lipid bilayer while HT is mainly in the aqueous core. In this way, the short half-life and low solubility in aqueous milieu of free HT and Cur can be overcome. However, conventional liposomes are somewhat unstable in the gastrointestinal tract (GIT). In fact, orally administered liposomes are subject to extreme pH conditions, to the action of enzymes and bile salts which greatly reduce their concentration in GIT [21]. Part of the vesicles is destroyed by the effect of gastric acid, with partial release of their payload. Into the small intestine, another fraction of liposomes undergoes micellization and degradation by intestinal surfactants and enzymes [22]. Only the vesicles that survive this step can penetrate the mucus layers, reach the intestinal epithelium, and can be absorbed as integral vesicles via the M cell-to-lymph pathway [21,23]. The destruction of the vesicular structure may not be an insurmountable obstacle for the absorption of hydrophobic molecules, which can also be mediated by mixed micelles originating from liposomes and bile salts [21]. However, it can be problematic if exposure to the aqueous environment of a particularly degradable load such as Cur is increased. On the other hand, the loss of the aqueous core is absolutely detrimental to water-soluble and highly labile molecules such as HT.

**Figure 1:**
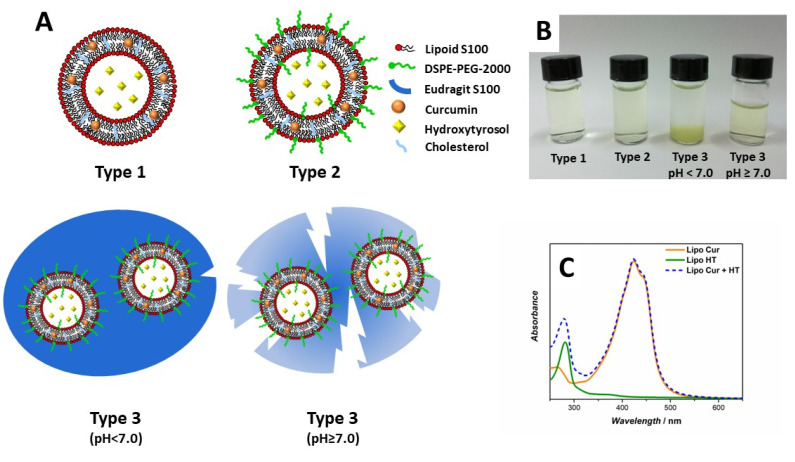
(**A**): Sketch illustrating the composition of the different types of liposomes tested. Type 1: LS100 (4 mg/mL) + Chol (10% mol); Type 2 LS100 (4 mg/mL) + Chol (10% mol) + 1,2-distearoyl-sn-glycero-3-phosphoethanolamine-N-[amino(polyethylene glycol)-2000] (DSPE-PEG-2000) (7% mol); Type 3: LS100 (4 mg/mL) + Chol (10% mol) + DSPE-PEG-2000 (7% mol) + EuS100 (0.625 g/g lipid). In all samples the initial concentrations of Cur and HT are 40 µM and 22 mM, respectively. The various elements are not to scale. (**B**): Picture showing Types 1–3 as prepared liposomes and Type 3 liposomes after EuS100 coating dissolution at pH ≥ 7.0. (**C**) UV–Vis spectra of Cur-, HT- and Cur/HT-loaded Type 2 liposomes upon transformation in mixed micelles.

In this work, the liposomes were adapted to the oral co-delivery of Cur and HT through modification with a matryoshka-like polymer barrier, consisting of an inner layer of polyethylene glycol 2000 (PEG-2000) and an outer coating of Eudragit S100 (EuS100) (Figure 1). EuS100 is an anionic copolymer based on methacrylic acid and methyl methacrylate, whose solubility is pH dependent. It is used in pharmaceutical formulations, resists gastric conditions (pH < 7.0) and dissolves above pH 7.0 in the upper bowel. The coating of PEG-2000 on the surface of liposomes is considered to be able to prevent enzymatic degradation, increase resistance to bile salts and also promote mucus-penetration as previously demonstrated [21,24,25,26]. The use of two polymeric coatings of PEG-2000 and EuS100 is an unprecedented strategy to adapt liposomes for the oral delivery of two polyphenols simultaneously, and has been devised to increase the residence time of intact liposomes in GIT and increase the intestinal stability of encapsulated Cur and HT [21]. Compared to other proposed delivery systems, this strategy should increase the oral bioavailability of Cur and HT and could increase the biological efficacy of the two polyphenols used in combination thanks to the possibility of reaching multiple biological targets at the same time [4,27].

## 2. Results and Discussion

### 2.1. Liposome Preparation and Characterization

Liposomes with different grades of complexity were prepared with the extrusion technique and fully characterized in terms of mean diameter, morphology, encapsulation efficiency (EE%), and loading capacity (LC%) of Cur and HT. All samples were made with Lipoid S100 (LS100) and cholesterol (Chol) with a molar ratio of 10:1. The Type 1 liposomes, used as control, had no polymeric coverage, the Type 2 liposomes had a PEG-2000 coating and the Type 3 liposomes also an additional coating of EuS100 (Figure 1A).

**Figure 2:**
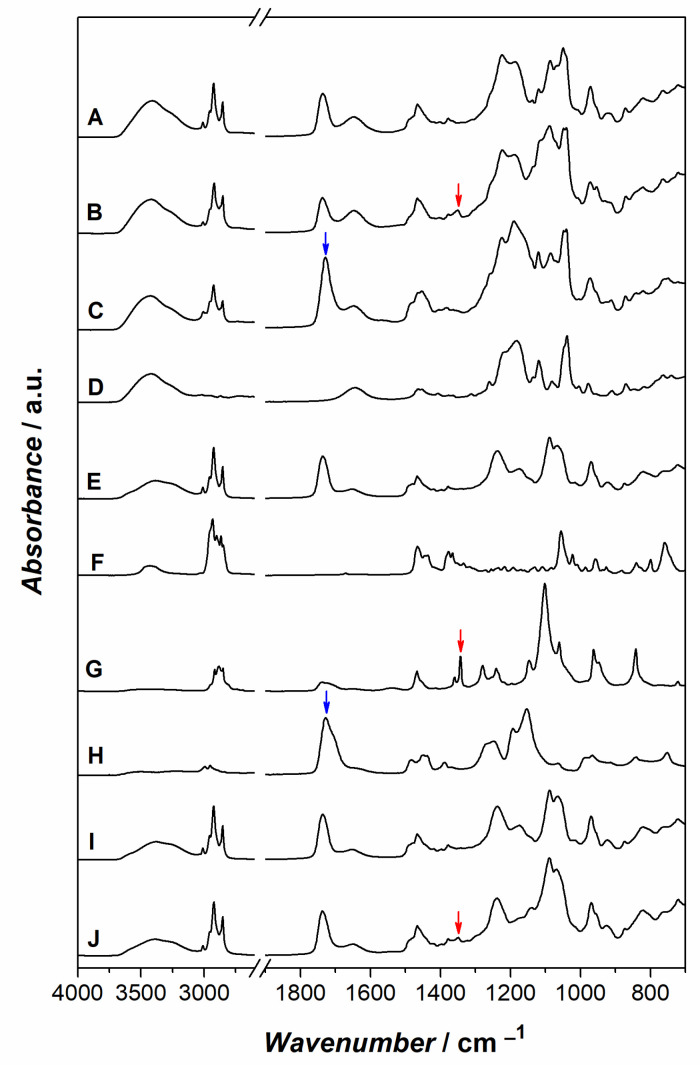
ATR-FTIR spectra of Type 1 liposomes (**A**), Type 2 liposomes (**B**), Type 3 liposomes (**C**), MES buffer (**D**), LS100 (**E**), Chol (**F**), DSPE-PEG-2000 (**G**), EuS100 (**H**), LS100/chol mixture 9:1 (**I**), LS100/chol/DSPE-PEG-2000 mixture 83:10:7 (**J**). See Materials and Methods for details on spectra acquisition. Arrows point out marker signals for DSPE-PEG-2000 (red) and EuS100 (blue).

The ATR-FTIR spectrum of Type 1 liposomes, shown in Figure 2 (trace A), presents the typical features of LS100 phospholipids (trace E) and MES buffer (trace D). Signals arising from Chol (trace F) are not detectable in Type 1 liposome spectrum. However, a possible loss of Chol during Type 1 liposome preparation can be ruled out since the spectral detection of Chol failed also in the case of a 9:1 LS100/Chol mixture cast on the ATR prism from a chloroform solution (trace I). The absence of signals clearly ascribable to Chol can be referred to the low Chol amount (10%), the low size (i.e., number of vibrating bonds) compared to LS100, and the lack of intense Chol signals in spectral regions where LS100 does not absorb. The infrared spectrum of Type 2 liposomes appears very similar to the one of Type 1 liposomes, with main contributions ascribable to LS100 and MES. Nevertheless, the presence of DSPE-PEG-2000 7% produces slight but detectable changes in the infrared spectrum. Specifically, the intense signal of DSPE-PEG-2000 at 1101 cm^−1^, due to the ether C-O stretching vibration of PEG (see trace G) clearly modifies the shape of the Type 2 liposomes spectrum in the 1200–1000 cm^−1^ region. Moreover, the sharp signal of DSPE-PEG-2000 at 1341 cm^−1^ (highlighted by red arrows) is well detectable in Type 2 liposomes spectrum. The ATR-FTIR spectrum of a LS100/chol/DSPE-PEG-2000 mixture (trace J) with the same molar ratio of Type 2 liposomes reproduces the same feature, corroborating the efficient incorporation of the pegylated lipid. The infrared spectrum of EuS100 (trace H) is dominated by the intense ester C=O stretching signal, ascribable to methacrylate. In agreement with the efficient coating of Type 3 liposomes (trace C) with EuS100, the ATR-FTIR spectrum of this preparation presents a characteristic very intense C=O band arising from superimposition of methacrylate and fatty acyl group vibration bands.

DLS analysis (Table 1) showed as liposomes of Types 1 and 2 loaded with Cur or HT were around 100 nm in diameter, compatible with the porosity of the polycarbonate membranes used during the phase of extrusion. Type 3 at pH < 7 has a micrometric size, following the inclusion of the vesicles in a polymer cluster of EuS100, while at pH ≥ 7, i.e., in the conditions in which the EuS100 coating dissolves, the average diameter of the liposomes returned to around 100 nm, as Types 1 and 2.

Cur and HT content in liposomes was estimated spectroscopically after sample micellization with LDAO detergent to remove scattering contribution (Figure 1C). The EE% and LC% values for Cur and HT, loaded individually or in combination in liposomes, appeared independent from the type of the carrier under consideration and therefore from the presence of the polymer coatings (Table 2). Cur is highly hydrophobic and tends to accumulate easily in the bilayer when added to the lipid blend. In previous works, we have determined the amount of Cur that settles in the bilayer without disturbing the structure and stability of the liposomes [8,9]. This allowed us to obtain high EE% values for the vesicles prepared in this work. On the contrary, HT is amphipathic and has a good solubility in water. It distributes between the core of liposomes and the outer aqueous solution. Thus, higher concentrations of HT were needed to force its encapsulation into the carriers. The small core volume of the liposomes compared to the external solution and the elevated concentrations of HT used explain the low values of EE% obtained. Nevertheless, the LC% values, which represent the amount of bioactive component that the carrier can effectively deliver, were higher for HT than Cur. The EE% and LC% values of HT in the Type 3 liposomes were lower than those recorded for Type 1 and Type 2 samples. This was probably caused by the vesicle covering process with EuS100, which involved a preliminary dilution of the sample and then several rinses (see paragraph 3.2), resulting in a loss of about 15% of the initially trapped HT. Finally, HT appeared to reduce slightly the EE% and LC% values of Cur when the two polyphenols are loaded into the vesicles at the same time. HT has an amphiphilic character and is evidently capable of partitioning at the phospholipid membrane and at the water-lipid interface [16,28], thus partially competing with Cur encapsulation. This would explain the slightly lower EE% value of Cur observed in liposomes containing both polyphenols.

**Figure 3:**
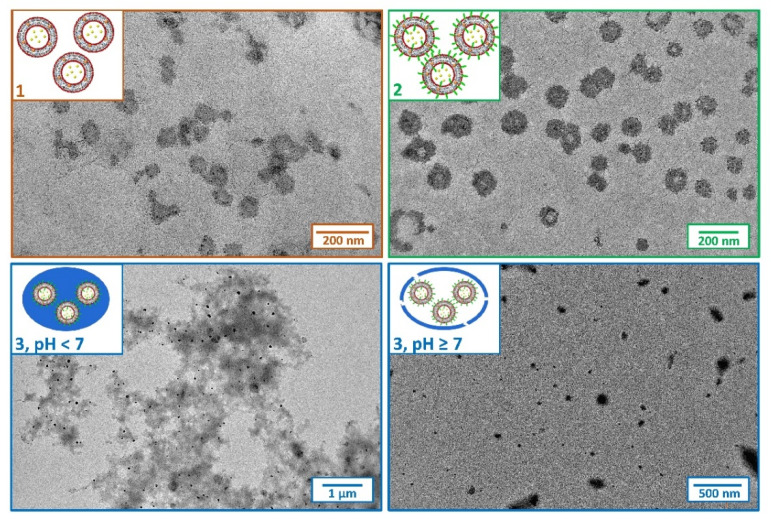
TEM images of Types 1–3 liposomes loaded with Cur and HT. The initial concentrations of Cur and HT are 40 µM and 22 mM, respectively. The sketches provide a qualitative description of the realized systems, and the dimensions of the different components are not to scale.

Transmission electron microscopy (TEM) images were collected to investigate the morphology and confirm DLS measurements. Figure 3 shows TEM images of the three types of liposomes containing Cur and HT simultaneously. Micrographs of Type 1 and 2 liposomes show nanoparticles with dimensions around 100 nm as expected, and with round-shaped morphology. In Type 3 liposomes under acidic conditions, it is possible to appreciate a micrometric cluster of spherical structures of about 100 nm enclosed in a polymer surrounding. This result confirms what was previously observed in the case of liposomes containing only Cur. The rapid pH jump to which EuS100 is subjected during preparation induces the precipitation of the polymer as aggregates enclosing liposomes [8,9]. At pH ≥ 7.0, the Eudragit coverage dissolves and free vesicles of about 100 nm were again observed. The process of precipitation and dissolution of the EuS100 clusters in the different pH conditions, with corresponding sequestration and release of the liposomes loaded with Cur and HT, is visually well observable in Figure 1B. In particular, it can be appreciated that at pH ≥ 7.0 there is a perfect redispersion of the vesicles in solution.

### 2.2. In Vitro Digestion

#### 2.2.1. Liposome Characterization during the In Vitro Digestion

**Figure 4:**
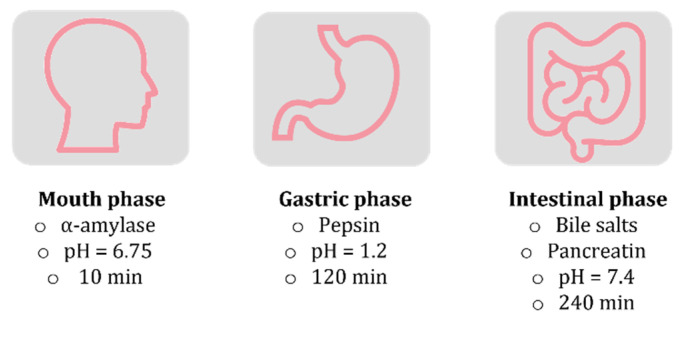
Enzymatic composition of the fluids, pH and incubation time characterizing each simulated in vitro digestive phase. Each digestive phase was carried out separately and at 37 °C. Liposomes/digestion fluids ratio was 1:3 *V/V*.

Liposomes (Types 1–3) were subjected to a simulated in vitro digestion composed of three separated steps: mouth, gastric and intestinal phases. The composition of the fluids, the pH and the incubation time that characterized each digestive phase are illustrated in Figure 4. In each phase, the diameter, the polydispersity index (PDI), and the ζ-potential of liposomes were evaluated (Table 3). The mouth phase was characterized by a pH = 6.75 and from a very short time of incubation (10 min). In these conditions, all the samples were stable from the size point of view and the ζ-potentials slightly changed. At this pH value, Eudragit polymer was insoluble and Type 3 liposomes remained embedded in polymer coverage.

**Figure 5:**
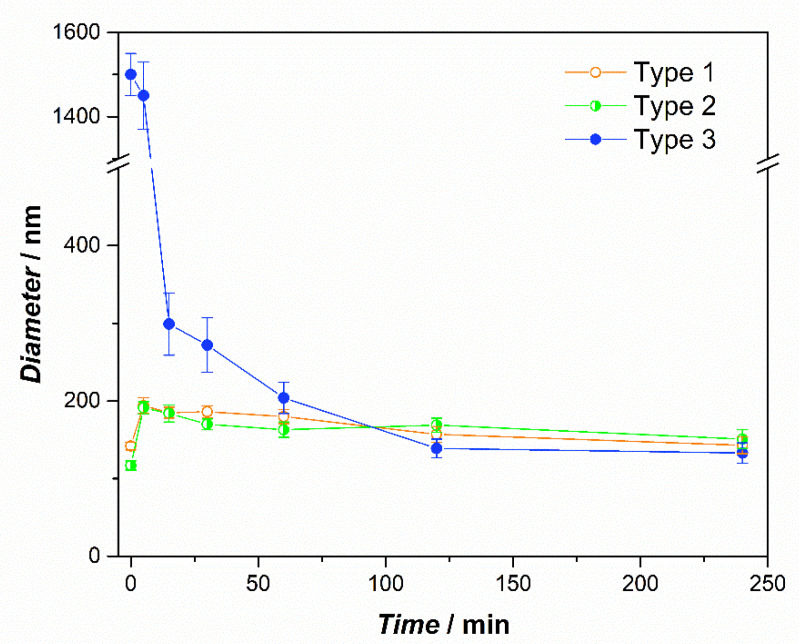
Mean diameter of Type 1–3 liposomes as a function of simulated intestinal digestion time. The error bars represent the standard deviations of the experiments (*n* = 3).

The gastric phase was characterized by strong acidic pH and lasted 120 min. In these conditions, the EuS100 polymer was still insoluble, and Type 3 liposomes remained unchanged. Moreover, the structure of Type 1 and 2 liposomes remained substantially unchanged in terms of mean size, despite the extreme acid conditions and the strong difference in osmotic pressure between the inside and the outside of the vesicles. This result has previously been observed for similar systems [29,30] and attributed to the fact that phospholipids cannot be hydrolysed in the stomach [31], and that cholesterol and phospholipids establish hydrogen bonds giving rise to a well-organized structure and with increased rigidity, which would improve the structural stability of liposomal membranes against gastric environmental stress [30,32]. The variations in the ζ potential in the gastric phase seem to indicate a certain instability of the formulations or even aggregation phenomena. In the intestinal phase, the pH reached slightly alkaline values (7.4) and the EuS100 polymer dissolved and released the liposomes, as demonstrated by the variation in the diameter from micrometric values to 124 nm for Type 3 system. The intestinal phase was the longest (240 min) and the variation in the diameter during this period was monitored in detail (Figure 5). In the case of Type 1 and 2 samples, it is possible to appreciate that their mean diameter increased during the first minutes of the digestion and then decreased. This is consistent with what has been observed by Liu and collaborators for similar vesicles [29], and may be due to the activity of the pancreatic enzymes which hydrolyse the phospholipids and to the interactions of bile salts with liposome components [29,30]. In Type 3 liposomes it was observed as a rapid decrease in the mean diameter caused by the dissolution of the shell of EuS100, with a following release of the vesicles. This can ensure a prolonged vesicle survival in GIT and a targeted drug release in the distal small intestine and the colon, where digestive enzyme and bile salt concentrations are low [33].

#### 2.2.2. HT Release during the In Vitro Digestion

The compounds entrapped into liposomes can be released through a mechanism of diffusion across the phospholipid membranes to an extent that depends both on the characteristics of the bilayer (thickness, surface area, transition temperature, fluidity, charge, etc.), and on the characteristics of the trapped molecules (size, polarity, charge, etc.) as well as those of the environment (temperature, pH, etc.) [34]. However, in the intestinal environment, the action of the bile salts and pancreatic enzymes can induce the loss of vesicular structure and then the transition of the liposomes into mixed micelles. This does not constitute a problem for hydrophobic compounds, which remain entrapped into the non-polar core of the micelles, but it brings to the complete and premature release of hydrophilic compounds such as HT [21].

The release of HT from each type of liposome was evaluated at the end of each digestive phase (Table 4). It was observed that Type 1 samples already released, significantly, the HT during the mouth and gastric phases. The HT exerts its bioactive function in the colon, therefore each release before the intestinal phase effectively represents a loss of the bioactive component. Type 2 liposomes showed lower releases in each digestive phase than Type 1 liposomes, but still not negligible. Type 3 vesicles, on the contrary, showed negligible mouth and gastric releases, which were found around 0–1%. This behavior is plausibly due to the polymeric coverage of EuS100 in which Type 3 vesicles are sealed and isolated from the external environment, as previously demonstrated. During the intestinal digestion phase, i.e., when the EuS100 coating dissolves due to the rise in pH, the release values of HT are brought to values similar to those of Type 2 liposomes. Under these conditions, Type 2 and Type 3 liposomes are identical in composition and structure and share the coating of only PEG-2000, thus release values for both are approximately 35%. On the contrary, Type 1 liposomes that have no polymer coating showed release values of approximately 55% in the same conditions. This highlights the role of PEG coating in determining lower releases from Type 2 and 3 samples in the intestinal phase, probably protecting the vesicles from premature micellization. For this reason, Type 3 liposomes appear to be the best candidates to protect their cargo of polyphenols within the lipid bilayer from premature release, and/or degradation in the hostile gastrointestinal environment [28].

**Figure 6:**
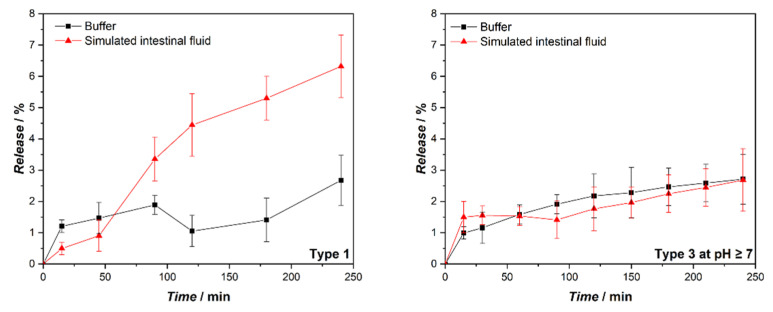
Comparison between the calcein release from liposomes of Type 1 and Type 3 (pH ≥ 7) when incubated in the simulated intestinal fluid (pH = 7.4) and K-phosphate buffered saline (PBS) solution (pH = 7.4). During the simulated intestinal digestion liposomes of Type 2 and 3 are equal since the EuS100 dissolves in these conditions. The error bars represent the standard deviations of the experiments (*n* = 3).

In order to better understand the effects of the PEG-2000 coating on HT release, liposomes of Type 1 and Type 3 at pH ≥ 7.0, respectively without and with PEG-2000 coating, were loaded with calcein, a strongly fluorescent hydrophilic molecule. Calcein is a model of polar hydrophilic drugs (Log P ≈ −4.02) and can be encapsulated in the aqueous compartment of liposomes. Its complete absence of membrane affinity justifies the low permeability of membrane to calcein and consequently, a mere passive calcein passage through the bilayer of liposomes can be assumed for intact liposomes [34]. Calcein-loaded liposomes were then subjected to incubation: 1) with simulated intestinal fluid containing bile salts and pancreatin in buffer at pH = 7.4; and 2) with the buffer alone, for the same time. The release of the calcein was then monitored over time (Figure 6). The release from Type 1 liposomes was higher in the presence of bile salts and enzymes, while they do not influence the release for Type 3 (pH ≥ 7) liposomes significantly. These results support the idea that liposomes without any polymeric coatings are structurally perturbed in the presence of simulated intestinal fluid, but do not undergo complete micellization by bile salts at physiological concentration. In this case, in fact, following the complete loss of the vesicular structure, the release of the calcein from liposomes would be almost complete. On the opposite, PEGylated liposomes better resist as intact vesicles, and the observed release is probably due mainly to the passive diffusion process. At pH = 7.4, Type 3 liposomes are equal to Type 2 liposomes since, in these conditions, the Eudragit dissolves and PEGylated liposomes are released. Therefore, in this test, Type 2 liposomes were not further investigated.

**Figure 7:**
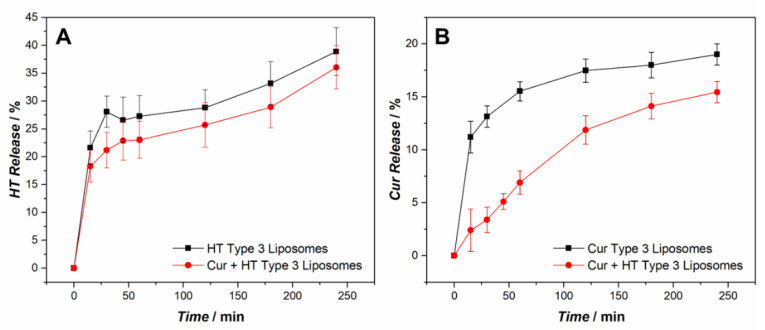
Release profiles of HT (**A**) and Cur (**B**) from Type 3 liposomes in simulated intestinal fluid (pH = 7.4). The black traces refer to liposomes containing only one polyphenol at a time, the red traces to liposomes containing both Cur and HT. The error bars represent the standard deviations of the experiments (*n* = 3).

The release profile of HT from Type 3 liposomes during intestinal digestion was also evaluated and is shown in Figure 7A. The HT is released rapidly during the first 30 min of the simulated digestion, and subsequently HT continued to be released but with a markedly reduced rate. The amphiphilic character determines the presence of polyphenol molecules at the water–lipid interface, which could be the first to be released in solution and faster. At the end of the simulated digestion about 35% of the HT is released, and there are no significant differences in behavior between the liposomes containing only HT and those containing both HT and Cur.

Similarly, the release of Cur from Type 3 liposomes during intestinal digestion was evaluated (Figure 7B). Cur is an extremely hydrophobic molecule, and a low release was expected for it. In fact, no signal attributable to Cur was recorded in the receiving solution. However, the initial concentration in liposomes decreased over time, and the release shown in Figure 7B was determined by the difference between the initial amount of Cur and that measured in liposomes in the time interval considered. A possible explanation for this result may come from previous studies which demonstrated that curcumin decomposed rapidly in buffer systems at neutral–basic pH conditions. In particular, Ying-Jan Wang and coworkers studied the degradation of curcumin in buffers at different pH values. They found that in phosphate buffer at pH 7.2 more than 90% of curcumin degraded within 30 min following an apparent first-order kinetics [35]. Therefore, the curcumin released by the liposomes in the simulated intestinal fluid at pH 7.4 would undergo a very rapid degradation. When Cur is present alone in liposomes (black trace in Figure 7B) after an initial burst effect, the release slows down to reach an apparent plateau at the end of digestion with a maximum release value of about 18%. The release or degradation phenomenon could quickly affect the Cur molecules most exposed to the external environment, and be slower for those buried deeper in the bilayer. In the presence of HT instead (red trace in Figure 7B) the release is slower, especially in the initial stages, and settles on slightly lower values than in the previous case at the end of the digestion. A more in-depth analysis would be needed to explain this different behavior, although a protective effect of HT on Cur could be hypothesized.

### 2.3. Antioxidant Activity

Liposomes grafted with PEG-2000 and entrapped into the EuS100 demonstrated to have the best performance under simulated in vitro digestion conditions. Once in the intestine, the EuS100 coverage dissolves. Here, PEGylated, Type 3 (pH ≥ 7) liposomes can interact with the cells and exert their antioxidant function. Thus, the antioxidant activity of liposomes loaded with Cur, HT, and both was evaluated through the 2,20-azinobis(3-ethylbenzothiazoline-6-sulfonic acid) radical cation (ABTS^•+^) decolorization assay and compared with the one observed for the 6-hydroxy-2,5,7,8-tetramethylchroman-2-carboxylic acid (Trolox), used as standard. Consequently, the antioxidant activity of the samples was expressed as Trolox Equivalent Antioxidant Capacity (TEAC). As a control, the activity of empty liposomes was estimated (Table 5).

The TEAC was evaluated in an absolute way normalizing for the concentration of the bioactive molecule under consideration, both in liposomes and in the proper solvent (2-(N-Morpholino)ethanesulfonic acid (MES) buffer for HT and MeOH for Cur).

As expected, empty liposomes showed a weak antioxidant activity imputable to the lipids used for the preparation of the vesicles. The absolute TEAC of the HT slightly decreased when encapsulated in liposomes compared to free HT in MES buffer, while no changes were encountered between Cur embedded in liposomes and in MeOH solution. This demonstrated the capability of HT and Cur to also act as scavengers of free radicals when embedded in the liposomes and virtually separated from the outer solution containing the ABTS. Absolute TEAC of liposomes containing both Cur and HT cannot be calculated, as the antioxidant activity cannot be referred to the concentration of two different species. However, in order to make a comparison between the antioxidant activity of Cur and HT when they are found alone or in combination within liposomes, Table 5 shows the “relative” values of the TEAC, i.e., expressed as μeq T/mL liposome suspension. In this way, the value obtained for HT/Cur-liposomes results as the sum of the TEAC of HT- and Cur-liposomes, once normalized for the tested concentrations of each species (see Section 3.6). Therefore, the action of HT and Cur embedded in liposomes against free radicals can be considered cumulative. In any case, it cannot be excluded that in vivo the two molecules may work in synergy. Furthermore, the different characteristics of these two polyphenols, such as for example their polarity, may allow them to reach different targets in vivo and exert peculiar biological effects (antioxidant, anti-inflammatory and antiproliferative) to prevent or fight different pathological factors [27].

### 2.4. Cytobiocompatibility of Liposomes

In order to characterize the biocompatibility of Type 3 (pH ≥ 7) liposomes, they were tested for cytotoxicity against intestinal Caco-2 cells through the 3-(4,5-dimethylthiazol-2-yl)-2,5 diphenyl tetrazolium bromide (MTT) assay. Cur, HT, and Cur/HT loaded liposomes were appropriately diluted in cell culture buffer to obtain the following final concentrations of bioactive components: [Cur] = 1.18 and 4.7 μM; [HT] = 10 and 40 μM. These Cur and HT concentrations were defined based on the previous literature showing an anti-oxidant effect of these bioactive molecules on Caco2-cells [7,31,32]. Empty liposomes were also tested to evaluate the inner toxicity of lipid carriers. Final lipid concentrations of 0.13 and 0.54 mg/mL were tested, almost equal to the actual lipid concentration in Cur and HT liposome used for MTT tests.

**Figure 8:**
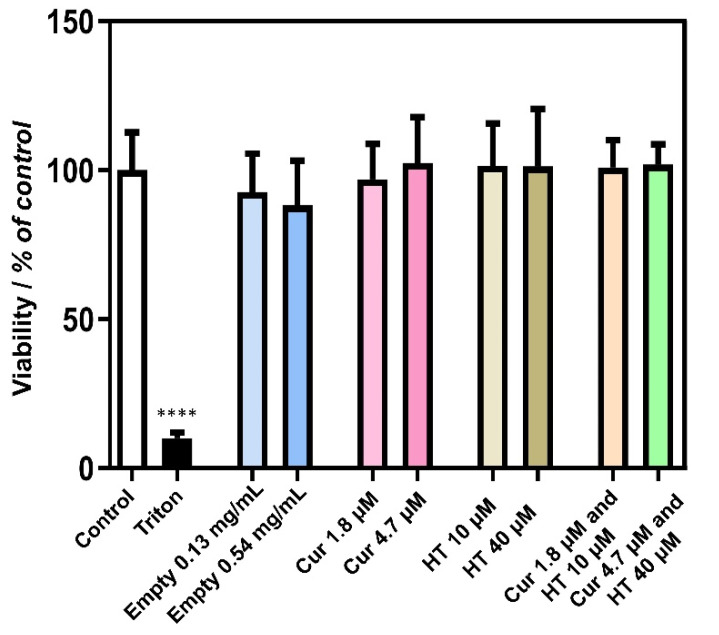
Effect of Type 3 (pH ≥ 7) liposomes on cell viability. Caco-2 cells were treated for 24 h with the noted concentrations of empty liposomes, Cur-liposomes (Cur), HT-liposomes (HT) and Cur/HT-liposomes (Cur/HT). Cell viability was determined as relative percent of viable cells of untreated control cells (control), put at 100%, by the MTT assay. Cells incubated with Triton X-100 (10% *v/v*) were used as positive controls. Data represent means ± SD of 14–16 samples per condition, obtained in two different experiments. **** *p* < 0.0001 Triton vs. control.

A 24-h incubation with Caco-2 cells showed no cytotoxic effects for all liposomal preparations, as compared to control untreated cells (Figure 8).

The concentrations used in the cytotoxicity assay are lower than those employed in the antioxidant assay (almost ten times for Cur and seven times for HT). However, HT was shown to reduce ROS production in Caco-2 cells, as determined by oxidized cholesterol [36] or acrylamide [37] at 10–40 μM. Moreover, we have previously shown that Cur-liposomes were active as anti-oxidant in Caco-2 at 2 μM curcumin [9]. This suggests that, when dealing with cells in vitro, the antioxidant efficacy can be reached with lower doses of those used in acellular assays, making our MTT assay sensitive to these effective concentrations.

## 3. Materials and Methods

### 3.1. Materials

All chemicals were purchased at the highest purity available and were used without further purification. Chloroform, ethanol, Sephadex G-50 medium resin, cholesterol, hydroxytyrosol, curcumin, calcein, the reagent grade salts for the 10 mM K–phosphate and 20 mM KCl (pH 7.0) buffer solutions (PBS Buffer) for the 10 mM 2-(N-Morpholino)ethanesulfonic acid and 20 mM KCl (pH 5.5) buffer solutions (MES Buffer), 100 mM Tris(hydroxymethyl)aminomethane (TRIS) buffer solutions, 2,20-azinobis(3-ethylbenzothiazoline-6-sulfonic acid) diammonium salt (ABTS), 6-hydroxy-2,5,7,8-tetramethylchroman-2-carboxylic acid (Trolox), dialysis tubing (high retention seamless cellulose tubing, MWCO 12400), *N,N*-Dimethyldodecylamine *N*-oxide (LDAO), potassium persulfate, Triton-X100 were purchased from Merck Italy (Merck Serono S.p.A., Rome, Italy). Enzymes (α-amylase from *Bacillus* sp., pepsin from porcine gastric mucosa, and pancreatin from porcine pancreas) and bile bovine (ox gall powder) for simulated in vitro digestion were from Sigma Aldrich (St. Louis, MO, USA). Lipoid S100 (approximately 100% soybean phosphatidylcholine) was from Lipoid (Lipoid GmbH, Ludwigshafen, Germany). Eudragit S100 was from Evonik (Evonik Industries AG, Darmstadt, Germany). Sterile filters of cellulose acetate (0.2 μm) were from Advantec. Polycarbonate Membranes (0.2 μm and 0.1 μm) and 1,2-distearoyl-sn-glycero-3-phosphoethanolamine-N-[amino(polyethylene glycol)-2000] (DSPE-PEG-2000) were purchased from Avanti Polar Lipids (Alabaster, AL, USA). All aqueous solutions were prepared by using water obtained from a Milli-Q gradient A-10 system (Millipore, Burlington, MA, USA).

### 3.2. Preparation of Liposomes

Liposomes of different grades of complexity were prepared with the extrusion method, as previously described [38], with the following compositions:

- Type 1: LS100 (4 mg/mL) + Chol (0.2 mg/mL, 10% mol);
- Type 2: LS100 (4 mg/mL) + Chol (0.2 mg/mL, 10% mol) + DSPE-PEG-2000 (1 mg/mL, 7% mol);
- Type 3: LS100 (4 mg/mL) + Chol (0.2 mg/mL, 10% mol) + DSPE-PEG-2000 (1 mg/mL, 7% mol) + EuS100 (2.5 mg/mL, 62.5% *w/w*).

Depending on the type of sample, the appropriate volumes of chloroform solutions of LS100, Chol, and DSPE-PEG-2000, were carefully blended in a round-bottom flask. For Cur-loaded liposomes, a methanolic solution of curcumin was added to the lipid blend to obtain a final concentration of Cur 40 μM (= 14.7 μg/mL). The organic phase was further evaporated under a gentle flux of nitrogen, followed by complete drying under vacuum conditions. Then, the lipid film was hydrated with MES Buffer and vortexed to obtain the vesicles. The dispersion was sonicated to reduce the dimensions of the vesicles and extruded to obtain a uniform and unilamellar dispersion. The extrusion was performed with a mini-extruder (AvantiPolar Lipids) first using polycarbonate membranes of about 200 nm (11 times), and then with membranes of 100 nm (11 times). The obtained samples were purified from unloaded curcumin through size exclusion chromatography using resin Sephadex-G50 medium. For HT-loaded liposomes, the lipid film was hydrated with an HT solution (22 mM = 3.4 mg/mL) in MES Buffer. Samples were purified from unloaded HT through dialysis (cut off 12,400 Da) overnight.

Calcein-loaded liposomes were prepared by hydrating the lipid film with a solution of calcein 40 mM in TRIS buffer (100 mM) pH 7.4. The samples were purified twice through size-exclusion chromatography using Sephadex-G50 resin.

Liposomes were encapsulated within EuS100 using a pH-driven method [8,9]. Briefly, 0.5 mL of liposomes were mixed with 0.5 mL of EuS100 in K-phosphate buffer (50 mM) pH = 8.0. The obtained solution was injected into 9 mL of acetic acid 0.1% under stirring. The precipitation of the EuS100 and the embedding of the liposomes in the cluster occur when the pH changes from basic to acid values. The polymer precipitate was washed three times with MES buffer to remove material eventually leaked during the coating. Vesicles for cell-based assays were sterilized with 0.45 µm sterile filters.

### 3.3. Characterization of Liposomes

The particle size and the PDI of liposomes and EuS100-liposomes were determined before and after each phase of the digestion by dynamic light scattering (DLS) analysis using a Zetasizer Nano ZS (Malvern Panalytical Ltd., Malvern, UK). The ζ-potential determination was performed using laser Doppler anemometry (Zetasizer Nano ZS, Malvern Panalytical Ltd., Malvern, UK) after dilution in distilled water (1:10).

UV–Vis absorption spectra of solutions and suspensions were acquired by means of a Cary 5000 (Agilent Technologies, Santa Clara, CA, USA) ultraviolet–visible, double-beam spectrophotometer. The resolution was set to 1 nm, and Hellma quartz cuvettes of 1 cm path length and suitable capacity were employed [39]. The amount of entrapped Cur was evaluated by exploiting its peak of absorption at 420 nm. To remove the scattering from the spectra, liposomes were transformed into mixed micelles upon the addition of the detergent LDAO (4% *v/v*). In the same way was evaluated the quantity of entrapped HT, but exploiting its peak of absorption at 280 nm. For comparison, the not encapsulated hydroxytyrosol was estimated in the dialysis buffer. For samples containing both HT and Cur, HT was evaluated after deconvolution of peaks at 280 nm to remove the Cur contribution.

Attenuated total reflectance Fourier transform infrared (ATR-FTIR) spectroscopy measurements of liposome formulations and standard compounds were acquired by means of a Perkin Elmer Spectrum One spectrophotometer equipped with a universal ATR accessory. The internal reflection element (IRE) was a three-bounce diamond microprism with a 4 mm diameter. The 3 µL of aqueous samples (liposome suspensions and buffer) were cast onto the ATR crystal and the water was left to evaporate by means of a gentle nitrogen stream. In the case of lipids (LS100, Chol and DSPE-PEG-2000) chloroform solutions were employed, and the use of the nitrogen stream was not needed since solvent evaporation was very fast. The ATR-FTIR spectra of dried samples were acquired at 4 cm^−1^ resolution. The spectrum of EuS100 at the same resolution was acquired placing the powder directly onto the ATR crystal and using a press to achieve a suitable contact with IRE and sample. For each spectrum 16 interferograms were recorded and averaged.

The encapsulation efficiency was evaluated through the following equation:

(1) $$EE\%=\frac{mg\;of\;entrapped\;bioactive\;components}{mg\;of\;initial\;bioactive\;components}\times 100$$

while the loading capacity:

(2) $$LC\%=\frac{mg\;of\;entrapped\;bioactive\;components}{mg\;of\;lipids}\times 100$$

Liposome morphology was investigated by transmission electron microscopy (TEM). TEM images were collected by a Jeol JEM-1011 microscope (Peabody, MA, USA) at an accelerating voltage of 100 kV and acquired by an Olympus Quemesa Camera (11 Mpx). Samples were prepared as previously reported [8].

### 3.4. Simulated In Vitro Digestion

Cur/HT-loaded liposomes were subjected to a simulated digestion following a reported protocol [29,40], slightly adapted to the purposes. Briefly, liposomes were incubated at 37 °C with the simulated digestion fluid under consideration at 1:3 *v/v* ratio. At the end, the mixture was ultracentrifuged (40,000 rpm, 3.5 h) to precipitate the liposomes and evaluate the release of the hydroxytyrosol into the supernatant by monitoring the absorbance at 280 nm. The composition of the simulated fluid and the incubation time varied according to the phase considered:

- Mouth phase: the simulated salivary fluid was composed of NaCl 8 mg/mL, α-amylase 1 mg/L, buffer KPi (2.5 mM), pH 6.75. The incubation was performed for 10 min;
- Gastric phase: the simulated gastric fluid is composed of pepsin 3.2 mg/mL, NaCl 0.03 M, pH 1.2. The incubation was performed for 120 min;
- Intestinal phase: the simulated intestinal fluid is composed of bile bovine 0.2 mg/mL, pancreatin 3.2 mg/mL, pH 7.4. The incubation was performed for 240 min.

### 3.5. Calcein Release Experiments

Calcein-loaded liposomes were subjected to the intestinal digestion with some modifications. Liposomes were mixed with the intestinal simulated fluid (1:3 *v/v*), put into a dialysis bag, and immersed into the intestinal simulated fluid in order to keep constant the composition of the fluid inside and outside the bag. The calcein released in the external fluid was evaluated by monitoring its emission fluorescence intensity (λ_ex_ = 495 nm, λ_em_ = 515 nm). The release was measured with the following equation:

(3) $$release\;\left(\%\right)=\frac{F_{t}}{F_{0}}\times 100$$

where F_0_ was evaluated by dispersing all the sample into the receiving buffer, after treatment of the liposomes with Triton-X100; F_t_ is the emission intensity at time t (min).

### 3.6. HT and Cur Release Experiments

The release of the bioactive compounds of interest from HT-, Cur-, and Cur/HT-loaded Type 3 liposomes during the intestinal digestion was monitored over time. Liposomes were mixed with the simulated intestinal fluid and put into a dialysis bag. The HT released was evaluated by monitoring its absorbance in the external fluid at 280 nm. After each measurement, the fluid was reintroduced into the medium. The release was measured with the following equation:

(4) $$release\;\left(\%\right)=\frac{A_{t}}{A_{0}}\times 100$$

where *A*_0_ was evaluated by dispersing all the sample into the receiving buffer, after treatment of the liposomes with LDAO; *A_t_* is the absorbance at time *t* (min).

Since Cur can not be estimated free into an aqueous medium, its release was evaluated by monitoring over time the decrease in the absorbance peak at 420 nm of the Cur in the liposomes into the dialysis bag:

(5) $$release\;\left(\%\right)=\frac{A_{0}-A_{t}}{A_{0}}\times 100$$

where *A*_0_ and *A_t_* are the absorbance values at 420 nm at time 0 and time *t* (min), respectively.

### 3.7. Antioxidant Activity Assay

Antioxidant activity was evaluated using the ABTS decolorization assay, suitably adapted for the analysis of liposome-embedded antioxidant samples as previously described [41]. The assay is based on the preventive formation of the radical cation ABTS^•+^, and on the subsequent evaluation of its neutralization by the antioxidant compound(s) present in the sample. The reaction is easily followed spectrophotometrically since ABTS^•+^ is a colored species, unlike the neutral form (hence the name of decolorization assay). The antioxidant-induced absorbance changes were recorded at 734 nm. Trolox was used as the standard antioxidant for the construction of suitable calibration curves. The antioxidant power of HT and Cur liposomes was expressed as the Trolox Equivalent Antioxidant Capacity. The antioxidant power of liposomes containing both HT and Cur was expressed as µeq Trolox/mL of liposome suspension. For comparison, the TEAC of HT in aqueous buffer and Cur in MeOH was also measured.

### 3.8. Cell Cultures and Cytotoxicity

The human intestinal epithelial cell line Caco-2 was grown as detailed before [9]. Caco-2 cells were plated at the number of 20,000 per each well of a 96-well plate and, on the following day, were incubated with Type 2 Cur-liposomes (1.18 and 4.7 μM Cur final concentration), HT-liposomes (HT 10 and 40 μM final concentration), Cur/HT-liposomes (Cur 1.18 and 4.7 μM and HT 10 and 40 μM final concentrations) and empty liposomes (lipids 0.13 and 0.54 mg/mL). Empty liposomes were diluted to obtain similar lipid concentrations of the loaded-samples (0.12–0.14 mg/mL and 0.49–0.56 mg/mL). Cells were then tested for viability after 24 h by the 3-(4,5-dimethylthiazol-2-yl)-2,5 diphenyl tetrazolium bromide (MTT) assay, as previously described [42]. The relative viability was calculated in respect to control untreated cells (considered as 100%). Cells treated with Triton-X-100 (10% *v/v*) were used as positive control.

### 3.9. Statistical Analysis

Statistical analyses were carried out by Prism Version 4, GraphPad Software Inc., La Jolla, CA, USA. Data were expressed as the mean ± SD. Multiple comparisons were based on one-way analysis of variance (ANOVA) with the Tukey’s post hoc test, and differences were considered significant when *p* < 0.0001.

## 4. Conclusions

In the present work, we developed polymer-modified liposomes that incorporate two antioxidant natural molecules, Cur and HT. These molecules have different polarities, and it was possible to load them simultaneously into the liposomes by exploiting the multicompartmentality of these lipid carriers. These nanosystems were intended for oral delivery applications and aimed to overcome the problems of instability and low bioavailability of these two natural molecules. To this end, liposomes were protected in an innovative way by two concentric polymeric coatings, the innermost of PEG-2000 and the outermost of EuS100. Unmodified liposomes were also made for comparison. Empty and antioxidant-loaded nanosystems were prepared and characterized in terms of physicochemical, morphological, drug incorporation and colloidal stability properties. Their behavior during in vitro digestion experiments revealed that the EuS100 outer coating was able to effectively protect the liposomes from mouth and gastric fluids, while dissolving in the intestinal one. Here, the second PEG-2000 coating grafted on vesicles appeared to confer greater strength to the liposome bilayer by minimizing the premature release of their bioactive cargo. Therefore, the proposed system seems to be able to deliver intact liposomes to the colon region characterized by near neutral pH, low enzymatic activity, low bile salt concentrations, long residence time, and slow secretion of mucus [33,43]. This should allow on the one hand a minimum degradation of the antioxidant load of Cur and HT, and on the other hand a maximum opportunity for their absorption. The experiments also showed that the antioxidant properties of the two molecules were conserved, and that there was no cytotoxicity of the proposed systems.

The proposed delivery systems should increase the oral bioavailability of both Cur and HT and could increase the biological efficacy of the two polyphenols used in combination, thanks to the possibility of reaching multiple biological targets at the same time. The different characteristics (first the polarity) of these two molecules should allow them to reach different compartments in vivo and exert peculiar biological effects to prevent or fight different pathological factors.

The herein developed polymer-modified liposomes appeared as promising candidates for oral delivery of Cur and HT, and also as a model for the delivery systems of molecules prone to degradation and characterized by low bioavailability.

## Figures and Tables

**Table 1:** DLS measurements of Types 1–3 liposomes loaded with Cur, HT, and Cur + HT.

Sample	Coating	Cur ^1^		HT ^1^		Cur + HT ^1^	
		Diameter (nm)	PDI	Diameter (nm)	PDI	Diameter (nm)	PDI
Type 1	none	115.5 ± 0.6	0.133 ± 0.009	109.6 ± 1.3	0.127 ± 0.011	114.5 ± 0.4	0.123 ± 0.010
Type 2	PEG-2000	100.6 ± 0.7	0.114 ± 0.012	108.7 ± 0.5	0.122 ± 0.014	102.6 ± 0.6	0.116 ± 0.008
Type 3, pH < 7	PEG-2000/ EuS100	>1000	N.D.	>1000	N.D.	>1000	N.D.
Type 3, pH ≥ 7	PEG-2000/ EuS100	114.3 ± 0.8	0.22 ± 0.02	110 ± 2	0.213 ± 0.018	113 ± 4	0.23 ± 0.03

^1^ The initial concentrations of Cur and HT are 40 µM and 22 mM, respectively. Data are expressed as mean ± SD (*n* = 3).

**Table 2:** EE% and LC% values of Cur and HT into liposomes (Types 1–3).

Sample	Cur ^1^		HT ^1^		Cur + HT ^1^			
	EE%	LC%	EE%	LC%	Cur-EE%	Cur-LC%	HT-EE%	HT-LC%
Type 1	81 ± 2	0.30 ± 0.02	2.5 ± 1.0	2.1 ± 0.9	69 ± 7	0.25 ± 0.02	1.9 ± 0.5	1.7 ± 0.3
Type 2	87 ± 3	0.32 ± 0.04	2.0 ± 1.1	1.6 ± 0.9	70 ± 9	0.26 ± 0.03	2.0 ± 0.8	1.7 ± 0.7
Type 3	87 ± 3	0.32 ± 0.04 ^2^	1.7 ± 0.9	1.4 ± 1.2 ^2^	70 ± 9	0.26 ± 0.03 ^2^	1.7 ± 0.6	1.5 ± 0.5 ^2^

^1^ The initial concentrations of Cur and HT are 40 µM and 22 mM, respectively. ^2^ The mass of the EuS100 polymer was not considered in the calculation. Data are expressed as mean ± SD (*n* = 3).

**Table 3:** Liposome characterization as prepared and after each phase of simulated digestion. Liposomes were loaded with Cur and HT (40 µM and 22 mM, respectively).

Sample		Diameter (nm)	PDI	ζ-potential (mV)
Type 1	As prepared	114.5 ± 0.4	0.123 ± 0.010	−1.7 ± 0.3
	After mouth digestion	114.9 ± 1.8	0.112 ± 0.005	−4.8 ± 0.2
	After gastric digestion	109.6 ± 1.3	0.15 ± 0.03	+9.7 ± 1.3
	After intestinal digestion	119.0 ± 1.0	0.156 ± 0.008	−19.6 ± 0.8
Type 2	As prepared	102.6 ± 0.6	0.116 ± 0.008	−9.3 ± 0.3
	After mouth digestion	108 ± 2	0.18 ± 0.03	−5.2 ± 0.9
	After gastric digestion	109.7 ± 0.5	0.18 ± 0.03	−0.6 ± 0.2
	After intestinal digestion	110 ± 2	0.20 ± 0.03	−14.2 ± 1.5
Type 3	As prepared	>1000	-	−20.9 ± 1.3
	After mouth digestion	>1000	-	−16.4 ± 2
	After gastric digestion	>1000	-	+0.8 ± 0.4
	After intestinal digestion	124 ± 3	0.23 ± 0.02	−25.3 ± 0.2

Data are expressed as mean ± SD (*n* = 3).

**Table 4:** %HT release at the end of the three phases of the digestion.

Sample	Release in Mouth Phase	Release in Gastric Phase	Release in Intestinal Phase
Type 1	(17.9 ± 1.5)%	(16 ± 2)%	(54 ± 4)%
Type 2	(12 ± 2)%	(9.5 ± 1.4)%	(36 ± 2)%
Type 3	(0.7 ± 0.2)%	(0.040 ± 0.007)%	(35 ± 3)%

Each digestive phase was carried out separately. Data are expressed as mean ± SD (*n* = 3).

**Table 5:** Antioxidant activity of Type3 (pH ≥ 7) empty, HT-loaded, Cur-loaded, and HT/Cur-loaded liposomes.

Sample	Cur (μM)	HT (μM)	TEAC (Absolute)	TEAC (μeq T/mL Liposome Suspension)
Empty liposomes	0	0	--	0.011 ± 0.003
HT-liposomes	0	280	0.90 ± 0.02	0.25 ± 0.06
Free HT ^1^	0	280	1.2 ± 0.2	
Cur-liposomes	36	0	3.19 ± 0.13	0.12 ± 0.05
Free Cur ^2^	27	0	3.25 ± 0.15	
HT/Cur-liposomes	32	229	--	0.29 ± 0.06

^1^ in MES buffer solution; ^2^ in MeOH solution. Data are expressed as mean ± SD (*n* = 3).

## Data Availability

Not applicable.
